# A visual model approach to extract regions of interest in microscopical images of basal cell carcinoma

**DOI:** 10.1186/1746-1596-8-S1-S36

**Published:** 2013-09-30

**Authors:** Ricardo Gutiérrez, Eduardo Romero

**Affiliations:** 1 CIM&LAB – Telemedicine Centre, Universidad Nacional de Colombia, Carrera 30 No. 45-03, Medicine Faculty, Building 471, Bogotá, Colombia

## Background

The virtual microscopy is a discipline that emulates the interaction between an expert with a microscopical sample upon a high resolution digital slide [1]. This type of technology is used for medical training and medical education, but so far it has been exclusively used in research environments because of the large computational requirements [2]. For instance, after digitizing *1cm2* of a physical slide at a level of ×20 magnification, the resulting virtual slide amounts to a 4GB [3], that must be processed, transmitted, explored and analyzed in real time. This picture can be even worsen since a typical laboratory takes hundreds of slides each week [4].

Overall, a typical pathologist does not explore the entire slide, but instead she/he focus her/his analysis on a few number of visual fields or regions of interest (RoI ). In consequence, recognition of RoI in microscopical images may be a potential source of knowledge in many diagnostic tasks. Such RoIs would introduce new learning paradigms that would be used in medical education, medical training and diagnosis assistance. In addition, a precise determination of such regions can highly reduce the computational and transmission charge of informative regions from a sample. However, automatic recognition of such regions is really a challenging task because of the inherent randomness of tissue’s cutting, color tissue properties and tissue orientation.

In spite of these difficulties, the pathologist efficiently recognizes regions of interest in several domains by fusing image and task dependent information into a unique framework. This paper proposes a novel automatic approach to recognize RoIs by emulating the processing of the human visual system (HVS), not only modeling the preattentional process but also integrating it with high level processes. Hence, this paper extends our previous work [5] by including structural information about the relationships between several objects and texture recognition as higher cortex functions. These processes are necessary to minimally perceive the core of a scene, just as it is carried out within the pathologist memory [6], and therefore, to identify relevant regions for diagnosis.

## Material and methods

### Experimental setup

The model was tested with a total of 115 histological microscopical fields of view of different types of basal cell carcinoma, sampled from 25 randomly chosen patients. Each biopsy was formalin-fixed and stained with Hematoxylin-Eosin dyes. Microscopical fields were digitized with a Nikon eclipse E600 system, through a coupled Nikon DXM1200 camera, and stored in JPEG format at a 1280 × 1024 resolution using microscope magnifications of ×4, ×10, ×20 and ×40. An expert pathologist, with at least five years of experience, selected the digitized fields of view and manually segmented relevant regions. We use 20 images to extract textons of size 32 × 32 pixels from RoIs and background for the object recognition task, and 95 images to test the entire algorithm.

### Method overview

**Figure 1:**
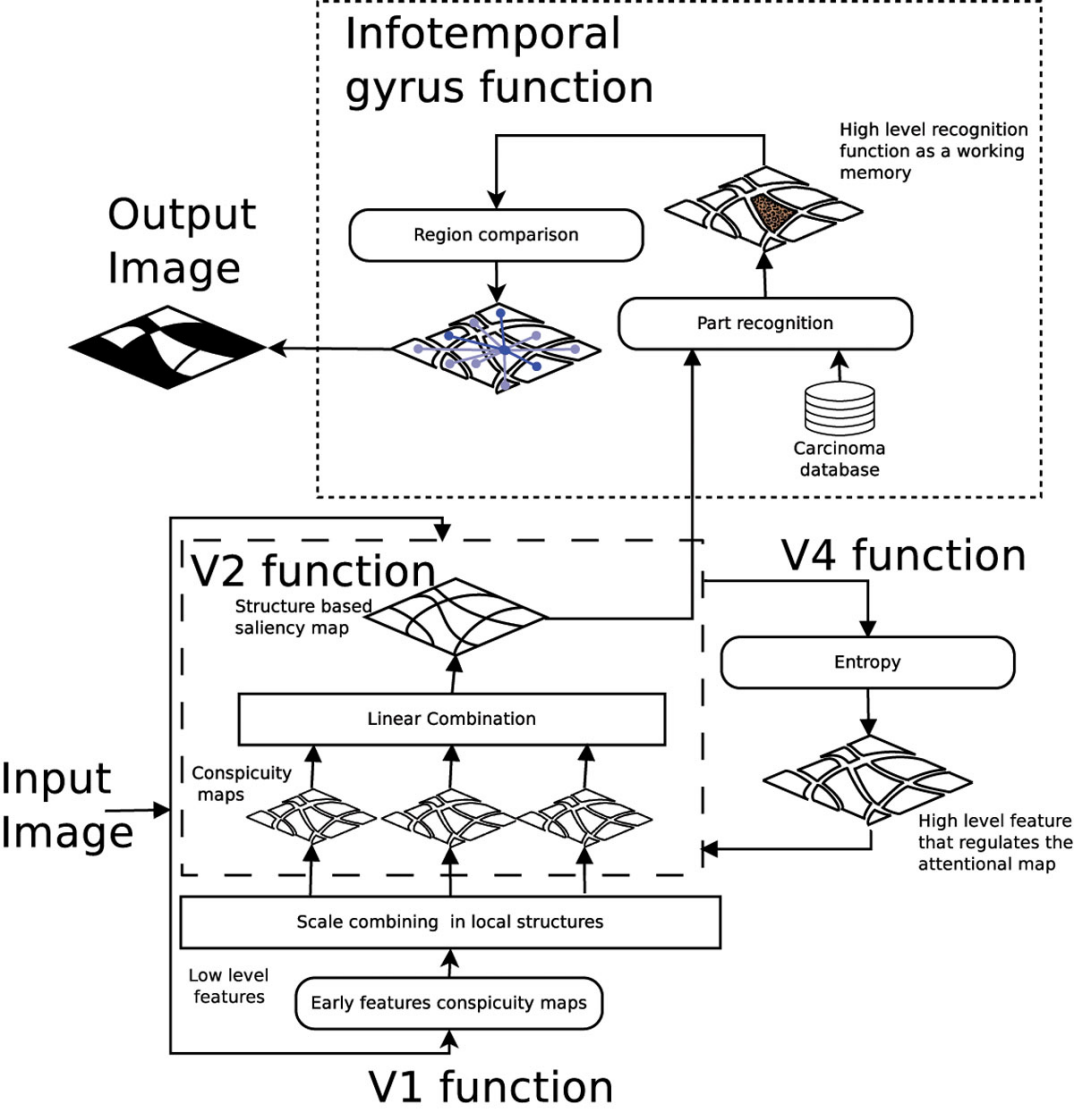
**The proposed model** The proposed model fuses the bottom-up and top-down information. Firstly, assigning a level of conspicuity based in a set of early characteristics, secondly, it gather together this information into simple shapes, thirdly, by regulating the saliency of each shape by the texture information as it is reported a relevant characteristic to diagnosis of cancer. Finally, our method uses the resulting saliency map to look for carcinoma structures using a database and a similarity measure over its neighborhood.

During a standard exploration of a histopathological sample, the pathologist integrates two types of information sources, namely, 1) the visual field content itself (“bottom-up” source), and 2) the knowledge involved in the specific task of diagnosis (“top-down” source). The HV S fuses together these sources using at least four different brain areas, namely, 1) the V1 cortex which assigns a local relevance to the visual input, 2) the V2 cortex which is responsible of gathering together these relevancies as simple shapes, 3) the V4 cortex which actively regulates the V2 excitation, and finally, 4) the inferotemporal gyrus which integrates the function of the previous areas by recognizing complex shapes and their purposes in the scene and probably by retrieving a slight image representation composed of a few objects actually recognized, the relations between them and the background, and basic information about the background texture [6]. The approach proposed herein models the function of the HV S in four steps, as shown in Figure 1. Firstly, it assigns a local relevance by integrating information from basic features as orientation, color and intensity at multiple scales, as previously described by Itti *et al*. [7] (V1 cortex function). Secondly, the model segments the image by taking into account the proximity and similarity between pixels and mixing up the conspicuity maps into the resulting regions (V2 cortex function), but also adding a map of the intrinsic structural disorder which models the specific task knowledge that regulates the attention over each structure (V4 cortex function) [5]. Thirdly, this intermediate map is used to feed a module that efficiently looks for basal cell carcinoma by comparing the pattern composition with a data base of carcinoma regions, starting with the highest attentional regions (inferotemporal gyrus function). Once the algorithm sets up the first carcinoma region within the image, the other carcninoma regions are defined as the most similar regions using an Euclidean metrics of the different basic features: color, entropy and image intensity.

Then, this intermediate map is used to feed a module that efficiently looks for basal cell carcinoma by comparing the pattern composition with a data base of carcinoma regions, starting with the highest attentional region (inferotemporal gyrus function). Once the algorithm sets up the first carcinoma region within the image, the other carcninoma regions are defined as the most similar regions using an Euclidean metrics of the different basic features: color, entropy and image intensity.

### Structure search and recognition

During a searching task of an undetermined number of targets, the pathologist determine whether she/he is familiar with the scene, using simple features as color and texture information, possibly recognizing some relevant objects and their relations [6,8]. If this is the case, she/he efficiently looks for the targets, following the saliency of the scene and then using the target contextual relationships [8,9]. Likewise, our proposal follows the most saliency levels of the map obtained in the local recognition step [5], that is say the third stage, when the system has determined a target. We extract then some textons from RoIs previously marked (object recognition) and some from the background (basic background features extraction) to discard irrelevant structures. A classical kNN algorithm was used, using the low level features captured before as input [10]. In case of finding a target, its relations with the other structures in the image are determined, comparing how different its internal features are. Formally, the distance between features (df ) within the target structure (st ) and the i-structure (si ) is defined as the Euclidean mean distance of the intensity features (I), orientation (O), color (C) and entropy(H) inside each structure, formally, 0 identifying similar structures as part of the searched targets using a simple threshold. Note that this algorithm addresses the intensive computations to a few structures, until it reaches the first target.

## Results and discussion

**Figure 2:**
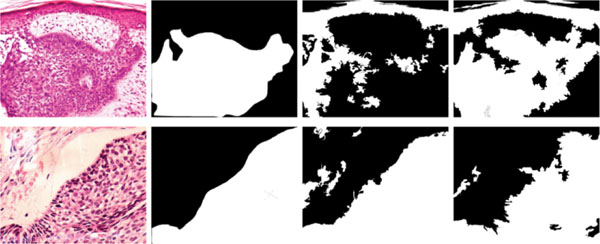
**Comparison between the previous work and the current one** From left to right, first column present the original images, the second one the ground truth marked by the pathologist, the third column presents the results obtained with Gutiérrez *et al*. (2011) strategy, and finally, the fourth column shows the results obtained with our method.

The method herein presented was compared with a previous one [5] that outperformed the state of the art. The model proposed uses as model a generic “top-down” information source [6], taking advantage of its relation with the context. The inferotemporal gyrus function improves the RoI selection, as illustrated in Figure 2. Note that the model proposed is more selective to the basal cell carcinoma structures since it is able to carefully identifies specific low level mixtures present in the first target reached and choose a clever similarity rule to define the discriminative threshold.

Also, the proposed model is more robust to distractors at several scales of magnification of the virtual slide. Our actual approach was tested with images at different scales of magnification, without any restriction at the training stage. The current method improves the results in specificity and reduces the variability of the results. These results are presented in the Table 1.

**Table 1:** Statistical results

	Mean (variance) [%]	
	Gutiérrez *et al.* (2011)	Ours

Sensitivity	86.6 (27.5)	80.8 (17.8)

Specificity	37.6 (23.7)	63.6 (19.0)

Sensitivity and Specificity results computed over several magnifications.

## Conclusions

This paper presented a novel methodology to find RoI based on the human visual system. This differs from our previous approach by the inclusion of a stage of region recognition and evaluation of inter-region similarity. These characteristics let us improve the RoI extraction since the selection criteria are modified by a knowledge database.

## List of abbreviations

HVS: Human Visual System; kNN: k-Nearest Neighbour; RoI: Region of Interest

## Competing interests

The authors declare that they have no competing interests.

## Authors' contributions

RG developed the algorithms and evaluated the results of the visual model. ER conceived the study, developed the fundamental ideas underlying this model, participated in the experimental design and was the director of the whole project. All authors read and approved the final manuscript.
